# Neoadjuvant Chemotherapy in Pregnant Patients with Cervical Cancer: A Monocentric Retrospective Study

**DOI:** 10.3390/curroncol29080450

**Published:** 2022-08-14

**Authors:** Federica Bernardini, Gabriella Ferrandina, Caterina Ricci, Anna Fagotti, Francesco Fanfani, Anna Franca Cavaliere, Benedetta Gui, Giovanni Scambia, Rosa De Vincenzo

**Affiliations:** 1 Dipartimento Scienze della Salute della Donna, del Bambino e di Sanità Pubblica, UOC Ginecologia Oncologica, Fondazione Policlinico Universitario A. Gemelli, IRCCS, 00168 Rome, Italy; 2 Dipartimento di Scienze della Vita e Sanita Pubblica, Università Cattolica Del Sacro Cuore, 00168 Rome, Italy; 3 Gynecology and Obstetric Department, Azienda USL Toscana Centro, Santo Stefano Hospital, 59100 Prato, Italy; 4 Dipartimento Diagnostica per Immagini, Radioterapia Oncologica ed Ematologia, UOC Radiodiagnostica Addominale, Fondazione Policlinico Universitario A. Gemelli, IRCCS, 00168 Rome, Italy

**Keywords:** cervical cancer, pregnancy, neoadjuvant chemotherapy, cesarean radical hysterectomy, tailored treatment

## Abstract

Background: To date, little and discordant data still exists on the management of cervical cancer (CC) during pregnancy. In this paper, we report our experience of the treatment of these patients analyzing the oncologic, obstetric, and neonatal outcomes. Methods: Between January 2010 and December 2021, 13 patients were diagnosed with CC during pregnancy. All patients underwent platinum-based neoadjuvant chemotherapy (NACT) and 11/13 patients underwent a cesarean radical hysterectomy (CRH). Results: All 13 patients were diagnosed with squamous-cell carcinoma, FIGO-2018 stage between IB2-IIIC1. The majority of patients had a partial (61.5%) or complete (15.4%) response to NACT. Most patients had a regular course of pregnancy and the obstetric complications observed were gestational diabetes mellitus in 23.1% and IUGR in 15.4% of cases. CRH was performed in the absence of major complications. Only 2 patients (15.4%) had disease recurrence and only 1 patient (7.7%) died of disease. All children are currently healthy. At birth, we observed mainly prematurity-related complications (38.5% respiratory distress syndrome and 7.7% neonatal jaundice) and only a case of congenital malformation (hypospadias). In our pediatric population, we reported a case of malignancy (acute myeloid leukemia). Conclusion: NACT seems to be safe and efficacious in controlling tumor burden during pregnancy. CRH following NACT appears to be feasible, avoiding repeated surgery and treatment delays. This approach is also reasonably safe from a maternal, obstetric, and neonatal point of view.

## 1. Introduction

Cancer in pregnancy is a rare event [1,2], although its incidence is expected to rise, due to the increase in the mean age at first pregnancy, and the increasing use of non-invasive prenatal tests (NIPTs) that can reveal the presence of asymptomatic tumors [3]. It is difficult to determine the exact incidence of cancer in pregnancy; in fact, in most countries, obstetric and cancer registries are not linked, and many national studies do not report information on the spontaneous abortion or termination of pregnancy often, thus altering the data. However, it is estimated that, in developed countries, the incidence of cancer in pregnancy is around 1 in 1000 pregnancies [4].

Cervical cancer (CC) is estimated to be the most frequent gynecological cancer in pregnancy (1.4–4.6 per 100,000), but, fortunately, more than 80% of cases are diagnosed at an early stage of disease [1,4,5,6,7].

In the past, the oncological treatment of CC has been considered incompatible with the evolution of pregnancy, but thanks to the exponential growth of knowledge in both oncology and obstetrics, it is now possible to guarantee a good fetal outcome without significantly altering the maternal prognosis. At present, it is clear that pregnancy does not worsen the prognosis of CC, and that pregnant patients have an outcome comparable to non-pregnant patients [8].

The treatment strategy in patients with CC in pregnancy should be chosen in relation to the tumor size, stage, gestational age at the diagnosis, as well as the patient’s wish to continue or terminate the pregnancy [9]. In this scenario, neo-adjuvant chemotherapy (NACT) has been shown to be a valid therapeutic option for patients with CC, allowing, on one hand, an acceptable control of neoplastic progression, and on the other, a delay of delivery until the fetus has reached a maturity that guarantees its viability outside the maternal uterus [7,10]. Despite the availability of ESMO guidelines (2019) on the management of cancer during pregnancy [5], these are currently based on limited data from expert opinions and a small number of cases [7,9,11,12,13].

The aim of our study is to report our experience in the treatment of CC during pregnancy with NACT followed by radical surgery (RS). We also analyze the oncologic, obstetric, and pediatric outcomes of these patients.

## 2. Materials and Methods

This monocentric, retrospective study was conducted according to the Declaration of Helsinki and was approved by the ethics committee of the Fondazione Policlinico Universitario Agostino Gemelli IRCCS (protocol number: 0002523/22).

The inclusion criteria were the following: age > 18 years, biopsy-proven CC, pregnancy, FIGO stage > IA2 and common histotypes (squamous, adenocarcinoma, and adenosquamous); exclusion features were rare histology and HIV positivity.

### 2.1. FIGO Staging

Patients were staged according to the 2018 FIGO stage. Pelvic MRI was performed to determine tumor size; stromal, myometrial and parametrial infiltrations; involvement of the vagina, ovaries, and pelvic peritoneum; possible infiltration of the recto-vaginal and vesico-vaginal septa; and status of pelvic and aortic lymph nodes.

A transvaginal ultrasound was performed to define the ultrasound features of the neoplasm (size, echogenicity, vascularization, stromal infiltration, parametrial infiltration, distance of the tumor from the internal uterine orifice, and infiltration of surrounding structures), and a colposcopic examination was performed to assess the infiltration of the vaginal fornix.

Following staging, a careful counseling about the treatment options, eventual maternal–fetal complications, and gestational age was conducted.

### 2.2. NACT

After signing the informed consent forms, chemotherapy (CT) treatment was started as soon as possible, but never before 14 weeks of gestation. The treatment administered was a platinum-based CT every 3 weeks. The number of cycles performed for each patient varied according to the gestational age at the start of treatment. No CT cycles were performed after 34 weeks of gestation. Weekly blood tests were performed to exclude bone marrow, liver, or kidney toxicity.

In order to define the most adequate surgical planning, a comprehensive clinical response evaluation by transvaginal pelvic ultrasound and MRI was performed.

### 2.3. Surgery

Delivery was performed by cesarean section (CS). The degree of radicality was tailored according to the clinical response: size of residual tumor, level of residual stromal infiltration, and LVSI. Type of radical hysterectomy (RH) was defined according to the Querleu–Morrow classification [14]. A systematic dissection of pelvic lymph nodes (LNs) was performed. Para-aortic LN dissection up to, at least, the inferior mesenteric artery was evaluated on a case-by-case basis in view of the risk of LN metastasis.

### 2.4. Fetal and Obstetrical Outcomes

Fetal wellbeing was monitored before and after each cycle of CT by ultrasound, while fetal biometry and fetal Doppler studies were performed monthly throughout the pregnancy. Fetal lung maturity was achieved through the prophylactic administration of 12 mg of betamethasone for two intramuscular administrations 24 h apart. The timing of delivery was individually assessed in relation to maternal and fetal wellbeing, as well as the response to NACT. Moreover, the delivery timing was established as at least 3 weeks after the last CT to avoid the nadir of hematologic toxicity and limit hemorrhagic and infective risks.

### 2.5. Placental Histology, Pathological Response after NACT, and Adjuvant Treatment

Placental histology was performed to exclude metastases.

A histological examination after RS was conducted in order to define the pathological response after NACT. Cervical residual tumor, LVSI, LNs metastases, and resection margins were evaluated. The pathological response was defined as follows: complete disappearance of tumor from the cervix (CR); residual disease with ≤3 mm stromal invasion, including in situ carcinoma (PR1); and persistent residual disease with >3 mm stromal invasion on surgical specimen (PR2) [15]. Women with positive LNs, parametrial involvement, positive margins, or PR2 underwent additional treatment (external-beam irradiation or chemoradiotherapy).

### 2.6. Data Record and Statistics

Medical records and follow-up data were collected retrospectively on an electronic database, REDCap (electronic data acquisition tools) hosted at the Fondazione Policlinico Universitario A. Gemellli, IRCCS. The data collected included age, gestational age at diagnosis, histology, FIGO stage, radiological examinations, type of treatment, pathological response to CT, surgical and pathological data, adjuvant therapy, children’s outcome and maternal follow-up.

Descriptive statistics were used to summarize the clinical–pathological features of the study population. Quantitative variables were described using the following measures: minimum, maximum, and median. Qualitative variables were summarized with absolute and percentage frequencies.

The disease-free survival (DFS) was calculated from the date of surgery to the date of relapse or the date of the last follow-up; overall survival (OS) was calculated from the date of diagnosis to the date of death or the date of the last follow-up.

Statistical Package for Social Sciences software version 25.0 (IBM Corporation) was used for the statistical analysis. All *p*-values were two-sided and a *p*-value < 0.05 was considered statistically significant.

## 3. Results

Between January 2010 and December 2021, 13 patients diagnosed with CC during pregnancy were treated at our institution.

Table 1 summarizes the features of patients as well as the treatment and clinical outcomes. The median age of the patients at diagnosis was 36 years old (range: 27–42). All patients were diagnosed with CC during the first or second trimester of pregnancy (median gestational age at diagnosis: 18 weeks, range: 5–25). All patients were diagnosed with squamous cell carcinoma (SCC).

The stage of disease, according to the FIGO 2018 classification, was IB2 for 2 patients (15.4%), IB3 for 3 patients (23.1%), IIA1 for 2 patients (15.4%), IIA2 for 1 patients (7.7%), and IIIC1 for 5 patients (38.4%).

### 3.1. NACT

All women received platinum-based NACT; the first treated patient (7.7%) (in 2010) received cisplatin 70 mg/mq every 3 weeks, 6 patients (46.15%) (between 2011 and 2016) received cisplatin 70 mg/mq and paclitaxel 135 mg/mq every 3 weeks, while 6 (46.15%) patients (after 2016) received carboplatin 5 AUC and paclitaxel 175 mg/mq every 3 weeks. The median number of cycles administered was 4 (range: 2–6).

The majority of patients had a clinical partial response (cPR) (n = 8, 61.5%) to CT; 2 patients (15.4%) achieved a complete response to NACT (cCR) and 2 patients (15.4%) had stable disease (cSD). Only 1 patient (7.7%) experienced the progression of disease (cPD) after 4 cycles of NACT, which required the discontinuation of treatment and anticipation of CS (Table 1).

The number of cycles performed for each patient varied according to the gestational age at the start of treatment.

### 3.2. Safety of Neoadjuvant Chemotherapy

CT was well tolerated by almost all patients, with mild symptoms, such as nausea or constipation and mild bone marrow toxicity.

The reduction in CT was required in 4 cases (30.8%): 1 patient (7.7%) presented G3 hepatotoxicity for which the paclitaxel dosage was reduced by 20%. Two patients (15.4%) presented G2 thrombocytopenia: in 1 case (7.7%) the dosage of carboplatin and paclitaxel was reduced, and in the other only the carboplatin dosage was reduced. Finally, 1 patient (7.7%) presented G3 anemia for which it was necessary to perform blood transfusion, reduce the dosage of carboplatin, and discontinue paclitaxel.

As far as the reactions to CT are concerned, 1 patient (7.7%) presented an allergic reaction to paclitaxel during the 2nd cycle of CT; the infusion of the drug was immediately stopped and corticosteroids were administered with clinical improvement. Paclitaxel administration was discontinued for the last two cycles. Despite the dose adjustment or continuation in monotherapy, no patient had to discontinue CT earlier than the established cycles.

### 3.3. Radical Surgery

RS was performed at the time of delivery for 11 patients (84.6%). The remaining 2 patients (15.4%) were managed by CS followed by RS; in the first, case one month after cesarean section, in the second after chemoradiation treatment performed for PD. Pelvic lymphadenectomy with or without an aortic lymphadenectomy was performed in all cases. Ten patients (76.9%) received bilateral salpingo-oophorectomy while 3 patients (23.1%) only underwent bilateral salpingectomy (Table 1).

Only 1 patient (7.7%) in the study population underwent laparotomic bilateral pelvic and aortic lymphadenectomy during the first trimester of pregnancy (11 weeks); the surgical procedure was performed without any intra- or post-operative complications. In a definitive histologic examination, LN metastasis was confirmed in 10/22 right pelvic and 1/25 left pelvic LN, whereas aortic LNs were negative for the localization of disease. The patient, following adequate counselling, was strongly motivated to preserve the pregnancy and accepted the proposed treatment.

The RS at the time of delivery was performed without major intraoperative complications in the majority of cases. Blood transfusion was required in 4 patients (30.8%) and an intraoperative hemorrhage occurred in 1 case (7.7%). Median blood loss during cesarean surgery and radical hysterectomy was 789 cc, and the median procedure duration was 140 min (Table 2).

Most of the patients had a regular post-operative course: we registered an early post-operative complication in 1 patient (7.7%) who presented obstructive urinary symptoms that resolved spontaneously a few weeks later. We only had 1 late post-operative complication (7.7%) in a patient who developed urge incontinence 3 months after surgery. Data on surgery, intra- and post-operative complications are presented in Table 2.

### 3.4. Histology

All placentas were sent for histological examinations and we registered the absence of placental metastases in all cases (Table 3).

As far as the residual tumor following surgery is concerned, 1 patient (7.7%) had no evidence of residual tumor and negative LNs, and 2 patients (15.4%) had a PR1 with negative LNs. Nine patients (69.2%) had PR2; of these, 6 (46.15%) had negative LNs and the remaining 3 (23.1%) had positive LNs. In 1 case (ID = 7, 7.7%), we observed PD during NACT, so CS was anticipated and RS was performed after chemoradiotherapy treatment (Table 1).

### 3.5. Adjuvant Treatment

In 3 cases (23.1%) in which patients had a CR or PR1 with negative LNs and margins, patients were referred for follow-up.

Six patients (46.15%) received adjuvant chemoradiotherapy. All these patients had a positive PR2 and LVSI: in 2 cases (15.4%), there were LN metastases, in 1 case (7.7%) there was positive margins, in 1 case (7.7%) both LNs and margins were positive, while in 2 cases (15.4%) LNs and margins were also negative.

Two patients (15.4%) underwent adjuvant radiotherapy; both patients had a PR2 and positive LVSI with negative LNs and margins.

One patient (7.7%) with PR2, positive LVSI, negative LNs and margins, who was offered adjuvant radiotherapy, refused treatment.

The patient who had PD (ID = 7, 7.7%) during NACT received combined chemoradiotherapy after CS; she had a microscopic residual cervical tumor at surgery (PR1) and was referred to follow-up.

The adjuvant treatment is reported in Table 1.

### 3.6. Pregnancy findings

The course of pregnancy was regular for most patients; we observed 2 cases (15.4%) of intrauterine growth restrictions, with fetal weight estimated at around 8° and 5°, respectively, and 3 patients (23.1%) were diagnosed with gestational diabetes mellitus, all treated with diet therapy (Table 3). We did not observe preterm labor, abruptio placentae, the premature rupture of membranes, or pre-eclampsia among our patients.

The median gestational age at cesarean delivery was 34.3 weeks (range: 31–36).

### 3.7. Pediatric Outcomes

To date, all children are alive and well. At birth, 5 babies (38.5%) required admission to the neonatal intensive care unit due to hyaline membrane disease and respiratory distress. During hospitalization, 1 of these children (7.7%) was also diagnosed with neonatal jaundice.

At 22 months of age, during surveillance controls, 1 child (7.7%) was diagnosed with M7 (megakaryoblastic) acute myeloid leukemia. The child underwent CT treatment and a bone marrow transplant and is currently disease free [16].

The same child at birth was diagnosed with hypospadias, the only case of congenital malformation in our case series (7.7%). Table 3 presents the obstetric and pediatric data of the study population.

### 3.8. Maternal Outcome

The median maternal follow-up was 40 months (range: 9 to 91). Two patients (15.4%) were lost to follow-up after 36 and 74 months, respectively. To date, 11 patients (84.6%) are alive without evidence of disease. Two patients (15.4%) had disease recurrence: the first had a pelvic relapse 12 months after adjuvant therapy and died after 2 years, the second had a pulmonary relapse, which was managed by a new line of CT and stereotactic radiotherapy. Despite treatment, the patient again had a further progression and started a new line of CT (Table 1). As far as OS, only 1 patient (7.7%) died of disease.

## 4. Discussion

To date, little and discordant data still exist in the literature on the management of CC in pregnancy, with considerable heterogeneity in terms of treatment, CT schedules, timing, and surgical procedures [7,9,11,12,13].

Our study showed that the treatment of CC during pregnancy by NACT and CRH was a successful choice in the management of these patients, characterized by excellent oncologic, obstetric, and pediatric outcomes.

In our case series, the majority of patients (61.5%) experienced a cPR and 15.4% a cCR. These data are in line with those reported in the literature, with a 7.2% complete response and 92.9% partial response [17].

In our population, only 2 patients (15.4%) had disease recurrence and, regarding OS, only 1 patient (7.7%) died of disease. Interestingly, the only patient who died of disease was the only one who received monochemotherapy with cisplatin and radical surgery following the cesarean section. The available, retrospective cohort studies confirm the excellent oncological outcomes of patients treated in pregnancy with NACT and CRH [12,13]. The outcomes of patients who decided to continue the pregnancy seems to be comparable to that of patients who chose to terminate it, and the prognosis of pregnant and non-pregnant patients seems to be similar [8].

CT is generally well-tolerated during pregnancy and is now considered a safe procedure [18,19]. Over the past three decades, the most widely used treatment has been cisplatin, either alone or in combination with other drugs [20,21]. Since 2010, many studies strengthened the evidence for the safety of carboplatin administration during pregnancy [22,23]. Experience with the use of taxanes in pregnancy is much more limited than with platinum derivatives. All the data available to date, obtained from retrospective studies and case reports, confirm the feasibility and safety of using taxanes during the second and third trimesters of pregnancy [24,25,26].

In our population, we observed mild to moderate bone marrow and liver toxicity; however, treatment discontinuation was never required and we observed the onset of allergic reactions only in one case. In the review conducted by Song et al. [13], moderate toxicity to NACT was described in 10 of 51 cases, including hematologic toxicity, drug intolerance, allergic reaction, nausea, and vomiting.

The main concern with the use of CT during pregnancy is its potential teratogenic effect on the fetus and the risk of miscarriage during the first trimester [27], as well as the risk of intrauterine growth restriction, prematurity, low birth weight, and bone marrow toxicity during the second and third trimesters [6,28]. In our case series, 2 patients (15.4%) presented with intrauterine growth restrictions, while 3 patients (23.1%) were diagnosed with gestational diabetes mellitus (GDM). To the best of our knowledge, no study analyzes the association between NACT and GDM. It is possible that corticosteroid prophylaxis administered before CT may increase the risk of GDM.

At birth, approximately 19% of newborns have adverse events [25], often related to prematurity whose incidence ranges from 48 to 61% [6,29]. The most frequent are respiratory distress syndrome and pathological jaundice. Additionally, in our experience, we observed 38% of infants with respiratory distress syndrome and 1 case (7.7%) of neonatal jaundice. A case of first-degree intraventricular hemorrhage is reported in the literature, and there is only one case of congenital malformation with an infant suffering from left-sided ventriculomegaly [26]. In our case series, we observed one case of hypospadias. The association between in utero exposure to endocrine disrupting chemicals and the occurrence of hypospadias has long been known [30]. Govers et al. also emphasized the importance of estrogen in penile development and how the loss of the estrogen signal is associated with hypospadias [31]. Obviously, these data do not allow us to hypothesize a causal relationship with in utero exposure to NACT.

Although the data concerning neonatal outcomes are reassuring, a case of severe hearing loss has been described in a child exposed in utero to cisplatin [32]. In our series, a child was diagnosed with acute myeloid leukemia at the age of two years. The child received a bone marrow transplant and is currently in remission of the disease [16]. Although the association between chemotherapies and the onset of secondary leukemia is known, it is not possible to establish a causal link between the two events. According to our knowledge, only another case of malignancy of children exposed in utero to cisplatin and paclitaxel has been described: the case of a five-year-old child, who was diagnosed with retroperitoneal embryonal rhabdomyosarcoma, with complete remission of the disease after two CT regimens [33]. This type of cancer mainly develops in the first decade of life and is closely associated with genetic factors or environmental exposures. Given the rarity of this phenomenon, however, it is not possible, also in this case, to define a causal relationship between the two events. Moreover, older data obtained from multicentric case–control studies examining the long-term outcome of children exposed to maternal cancer or cancer treatment, show that the cognitive and cardiac development and overall health of these children is not impaired during the early stages of childhood [29]. On the contrary, prematurity is frequently associated with impaired cognitive development [34].

In our series, regarding the long-term outcomes in children, all children were well, with regular cognitive development, and, in particular, the first girl (FUP 136 months–11.3 years) had a normal growth and she had her first period, confirming a regular ovarian function.

Our therapeutic choices have changed over time in relation to the experience gained in the management of our patients, the growing scientific experience, until the availability of official guidelines. Since most data in the literature are based on the use of cisplatin, we decided to treat our first patient in 2010 with a monochemotherapy of cisplatin at the standard dosage of 75 mg/mq. Growing evidence of the use of taxanes during pregnancy and our experience with disease recurrence following the use of platinum-based monochemotherapy led us to choose combination chemotherapy with cisplatin and paclitaxel for the following patients, initially at a reduced dose of paclitaxel (135 mg/mq) and later a full dose (175 mg/mq). Subsequently, the episode of acute myeloid leukemia onset in the son of one of our patients and the first piece of evidence of ototoxicity after cisplatin administration led us to modify the treatment with carboplatin 5 AUC and paclitaxel, which remains the preferred scheme.

Surgery also plays a crucial role in the management of patients with CC during pregnancy. The presence of LN metastases was documented in approximately 20% of patients with stage IA2 to IIA CC [35]. Staging lymphadenectomy is not recommended beyond 22 weeks of gestation, and its role in patients with FIGO stage IB3 is still controversial [5]. In our experience, we performed lymphadenectomy during pregnancy only in one case; this patient was diagnosed in early pregnancy and radiological examinations were strongly suggestive of LN involvement. The surgical procedure was performed without early or late complications, and fetal wellbeing was documented by an ultrasonographic examination. During a definitive histologic examination, LN metastasis was confirmed in pelvic lymph nodes bilaterally, whereas aortic lymph nodes were negative. In the study conducted by Favero et al., laparoscopic lymphadenectomy was performed in 18 patients. All the procedures were successfully completed in the absence of surgery-related maternal or fetal mortality or morbidity and without the need for laparotomy conversion. In 16% of cases, the lymph nodes were positive [36].

In CC, surgery also has a decisive role at the time of delivery. CRH not only prevents the need for a second anesthesia, but also avoids further delays in surgical treatment. The first experience of CRH dates back to 1958, when Brunschwig performed a CS, immediately followed by radical hysterectomy and pelvic LN dissection [37]. Combining RS with CS makes the procedure technically more complex due to the size of the gravid uterus limiting access to the surgical field. In addition, the procedure is associated with a higher rate of blood loss, intra-operative hemorrhage, and blood transfusions [38]. Despite this finding, the rate of other operative and post-operative complications appears to be comparable to that of a non-pregnant radical hysterectomy or with the fetus in situ [39], as does the surgical mortality rate [40]. In our experience, 11 of 13 patients (84.6%) performed CRH. Additionally, in our case series, we received a high rate of intra- or post-operative transfusions (45%), and 1 case (7.7%) of intraoperative hemorrhage. Moreover, from the currently available data, it is not possible to have a differentiated estimation of blood loss during the phases of CS and radical hysterectomy. In 2 cases (15.4%), we had an onset of urinary complications: in the first case, the patient had obstructive urinary symptoms resolved spontaneously following a few weeks; in the second case, the patient presented urge incontinence three months following surgery. However, both urinary complications are related to the type of RS performed, rather than to the timing at delivery.

The strength of our study is undoubtedly the sample size, since, to the best of our knowledge, there are no monocentric clinical studies with a larger and detailed study population. The main limitation is represented by the retrospective nature of the study. Another weakness of the study is also the administration of CT regimens that were not the same for all patients, which may have influenced the oncological outcome.

## 5. Conclusions

In a limited case series, our experience provided documented and supported evidence of the feasibility of a systematic treatment approach for women diagnosed with CC in pregnancy.

NACT with carboplatin and paclitaxel has proven to be both safe and efficacious from a maternal and fetal point of view, according to the available literature and our data. CRH following NACT seemed to be feasible in our experience as well, in order to avoid repeated surgery and treatment delays in these patients. This approach is also acceptably safe from a neonatal point of view, according to both our data and the available literature. Further studies are currently needed to support this evidence, as well as data concerning maternal and neonatal long-term follow-ups.

## Figures and Tables

**Table 1:** Clinical and pathological features of the study population.

Case ID (Age)	Gestational Week at Diagnosis	Histology	FIGO Stage 2018	Tumor Size (mm)	NACT	Response to CT	Tumor Size after NACT (mm)	Treatment	Pathological Response	Adjuvant Treatment	Recurrence	Maternal Outcome	FUP Month
1 (38 y)	18	SCC	IIA1	40	4 x cisplatin 75 mg/mg q 21	PR	12	(1) NACT (2) CS (34 w) (3) RH + BSO + PLND 1 month later	pR2 LVSI+ N− Margins+	RT-CT + BRT	Yes	DOD	31
2 (42 y)	13	SCC	IIA1	25	5 x cisplatin 75 mg/mg + paclitaxel 135 mg/mq q 21	PR	16	(1) NACT (2) CS + RH + BSO + PLND (31 w)	pR2 LVSI+ N− Margins−	RT	No	Alive	36
3 (34 y)	18	SCC	IB2	41	1 x cisplatin 75 mg/mg + paclitaxel 135 mg/mq q 21 3 x cisplatino 75 mg/mg + paclitaxel 105 mg/mq q 21	CR	13	(1) NACT (2) CS + RH + BSO + PLND (34 w)	pR0 LVSI− N− Margins−	No	No	Alive	74
4 (34 y)	25	SCC	IIIC1	70	2 x cisplatin 75 mg/mg + paclitaxel 135 mg/mq q 21	PR	40	(1) NACT (2) CS + RH + BS + PLND (35 w)	pR2 LVSI+ N− Margins−	RT-CT	No	Alive	91
5 (36 y)	20	SCC	IB3	47	4 x cisplatin 75 mg/mg + paclitaxel 135 mg/mq q 21	PR	31	(1) NACT (2) CS + RH + BSO + PLND (34 w)	pR2 LVSI+ N− Margins−	RT-CT + BRT	No	Alive	67
6 (37 y)	15	SCC	IIIC1	50	4 x carboplatin 5AUC + paclitaxel 175 mg/mq q 21	PR	41	(1) NACT (2) CS + RH + BSO + PLND (34 w)	pR2 LVSI+ N+ Margins−	RT-CT	No	Alive	46
7 (33 y)	23	SCC	IIIC1	80	3 x carboplatin 5AUC + paclitaxel 175 mg/mq q 21	PD	78	(1) NACT (2) CS (32 w) (3) RT-CHT (4) RH + BSO + PLND + ALND 4 months after	pR1 LVSI− N− Margins−	No	Yes	Alive	41
8 (41 y)	12	SCC	IB3	53	1 x carboplatin 5AUC + paclitaxel 175 mg/mq q 21 3 x carboplatin 4AUC + paclitaxel 135 mg/mq q 21	CR	0	(1) NACT (2) CS + RH + BS + PLND (34 w)	pR0 LVSI− N− Margins−	No	No	Alive	40
9 (27 y)	23	SCC	IB3	49	2 x carboplatino 4AUC + paclitaxel 175 mg/mq q 21 1x carboplatin 4AUC	SD	49	(1) NACT (2) CS + RH + BS + PLND (36 w)	pR2 LVSI+ N− Margins−	Refused	No	Alive	36
10 (35 y)	23	SCC	IIA2	49	2 x carboplatin 5AUC + paclitaxel 175 mg/mq q 21	SD	45	(1) NACT (2) CS + RH + BSO + PLND (34 w)	pR2 LVSI+ N− Margins−	RT + BRT	No	Alive	23
11 (36 y)	13	SCC	IIIC1	50	4 x carboplatin 5AUC + paclitaxel 175 mg/mq q 21 1 x carboplatin 4 AUC	PR	34	(1) NACT (2) CS + RH + BSO + PLND (36 w)	pR2 LVSI+ N+ Margins−	RT-CT + BRT	No	Alive	20
12 (35 y)	5	SCC	IIIC1	37	6 x carboplatin 5AUC + paclitaxel 175 mg/mq q 21	PR	31	(1) PLND + ALND (11 w) (2) NACT (3) CS + RH + BSO (34 w)	N+ pR2 LVSI+ Margins+	RT-CT + BRT	No	Alive	15
13 (39 y)	21	SCC	IB2	36	1 x carboplatin 5AUC + paclitaxel 175 mg/mq q 21 1 x carboplatin 4 AUC + paclitaxel 175 mg/mq	PR	15	(1) NACT (2) CS + RH + BSO + PLND (35 w)	pR1 LVSI− N− Margins−	No	No	Alive	9

ALND = aortic lymph node dissection; BRT = brachytherapy; BS = bilateral salpingectomy; BSO = bilateral salpingo-oophorectomy; CT = chemotherapy; CR, complete response; CS = cesarean section; DOD, death of disease; FIGO, International Federation of Gynecology and Obstetrics; FUP = follow-up; NACT = neoadjuvant chemotherapy; PD, progressive disease; PLND = pelvic lymph node dissection; PR = partial response; pR0 = complete disappearance of tumor in the cervix; pR1 = residual disease with ≤3 mm stromal invasion, including in situ carcinoma; pR2 = persistent residual disease with >3 mm stromal invasion on surgical specimen; RH = radical hysterectomy; RT = external-beam radiotherapy; SCC = squamous cell carcinoma; SD = stable disease.

**Table 2:** Characteristics of surgery performed during pregnancy and intra- and post-operative complications.

Case	Treatment	Time	EBL	Intra-Operative Complications	Post-Operative Complications
1	CS	70	400	No	No
2	CS + RH + BSO + PLND	140	2100	Hemorrhage	No
3	CS + RH + BSO + PLND	105	400	No	
4	CS + RH + BS + PLND	146	700	No	Transfusion
5	CS + RH + BSO + PLND	189	1000	Intraoperative transfusion	No
6	CS + RH + BSO + PLND	208	1300	Intraoperative transfusion	Obstructive urinary disorders
7	CS	75	700	No	No
8	CS + RH + BS + PLND	199	500	Intraoperative transfusion	No
9	CS + RH + BS + PLND	162	400	No	No
10	CS + RH + BSO + PLND	185	400	No	No
11	CS + RH + BSO + PLND	100	600	No	No
12	PLND + ALND CS + RH + BSO + PLND	96 203	50 1000	No No	No Urge incontinence
13	CS + RH + BSO + PLND	290	1500	Intraoperative transfusion	No

ALND = aortic lymph node dissection; BS = bilateral salpingectomy; BSO = bilateral salpingo-oophorectomy; CS = cesarean section; EBL = estimated blood loss; PLND = pelvic lymph node dissection; RH = radical hysterectomy.

**Table 3:** Obstetric and pediatric outcomes of the study population.

Case	Obstetric Outcome	Gestational Age at Delivery, w	Neonatal Weight, g	Placental Weight, g/Status	Sex	Apgar Score	Child Outcome	Current Health Status	FUP Month
1	Well	34 + 1	1950	380/M-	F	8/9	Well	Alive	136
2	Well	31 + 1	1520	390/M-	F	5/7	RDS	Alive	36
3	Well	34 + 2	2485	637/M-	M	4/8	RDS	Alive	74
4	Well	35 + 1	2450	555/M-	M	8/9	AML, Hypospadias	Alive	89
5	GDM	33 + 6	1995	412/M-	F	9/10	Well	Alive	64
6	GDM	34 + 2	2130	410/M-	F	9/10	Well	Alive	42
7	Well	31 + 5	1780	350/M-	F	6/8	RDS	Alive	38
8	IUGR	33 + 6	1860	414/M-	M	7/9	Well	Alive	37
9	Well	35 + 5	2545	520/M-	M	9/10	Well	Alive	34
10	Well	35 + 0	2435	357/M-	F	9/10	Well	Alive	21
11	IUGR	36 + 0	2300	607/M-	M	8/9	Well	Alive	15
12	GDM	34 + 5	2460	450/M-	F	9/9	RDS Neonatal jaundice	Alive	11
13	Well	35 + 1	2815	500/M-	M	7/7	RDS	Alive	8

AML = acute myeloid leukemia; GDM = gestational diabetes mellitus; IUGR = intra-uterine growth restriction; M = metastasis; RDS = respiratory distress syndrome.

## Data Availability

Medical records and follow-up data were collected on an electronic database, REDCap (electronic data acquisition tools) hosted at the Fondazione Policlinico Universitario A. Gemellli, IRCCS.
